# Oxidative Stress and Cardiovascular Complications in Chronic Kidney Disease, the Impact of Anaemia

**DOI:** 10.3390/ph11040103

**Published:** 2018-10-11

**Authors:** Faisal Nuhu, Sunil Bhandari

**Affiliations:** 1 School of Life Sciences (Biomedical), University of Hull, Cottingham Rd, Hull HU6 7RX, UK; 2 Hull York Medical School & Department of Renal Medicine, Hull and East Yorkshire NHS Hospital Trust, Hull HU3 2JZ, UK; Sunil.Bhandari@hey.nhs.uk

**Keywords:** oxidative stress, anaemia, cardiovascular disease, chronic kidney disease, IV iron therapy

## Abstract

Patients with chronic kidney disease (CKD) have significant cardiovascular morbidity and mortality as a result of risk factors such as left ventricular hypertrophy (LVH), oxidative stress, and inflammation. The presence of anaemia in CKD further increases the risk of LVH and oxidative stress, thereby magnifying the deleterious consequence in uraemic cardiomyopathy (UCM), and aggravating progression to failure and increasing the risk of sudden cardiac death. This short review highlights the specific cardio-renal oxidative stress in CKD and provides an understanding of the pathophysiology and impact of uraemic toxins, inflammation, and anaemia on oxidative stress.

## 1. Introduction

Oxidative stress refers to an imbalance in the reactive oxygen species (ROS) production/degradation ratio. ROS such as superoxide anions (O_2_^•^), hydrogen peroxide (H_2_O_2_), and hydroxyl radicals (OH^•^) (by-products of mitochondrial respiration) are important for cell signalling and immune response [1,2]. Inappropriate or excess generation of ROS can lead to deleterious cellular consequences including damage to DNA, proteins, and cell membrane lipids [3]. To limit these damaging effects, cells are equipped with endogenous capacity comprising enzymatic and non-enzymatic components to detoxify excess ROS. The latter consist of reductants such as reduced glutathione (GSH), vitamin C, beta carotenes, and vitamin E. GSH can deactivate ROS through its oxidation to the oxidised form (GSSG). Vitamin E, on the other hand, protects the cell membrane by reacting with lipid radicals. The enzymatic components consist of superoxide dismutase (SOD), glutathione peroxidase (GPx), glutathione reductase (GR), and catalase [4]. These enzymes together catalyse the dismutation of O_2_^•^ into water. Ineffective antioxidant capacity or excess generation of ROS is implicated in the development and progression of renal and cardiovascular diseases [5,6].

The association between renal and cardiac oxidative stress in chronic kidney disease (CKD) is poorly defined, and the impact of anaemia on the oxidant status of the kidney and heart is not completely characterized. In addition, many of the clinical studies in uraemic patients are limited to systemic oxidative stress, creating an information vacuum on cardiac oxidant status that must be filled by studies using experimental uraemic cardiomyopathy (UCM) models. Moreover, given that iron deficiency anaemia in CKD may exacerbate oxidative stress and worsen the poor cardiovascular outcome, how can parenteral iron therapy which is employed in current clinical practice modify oxidative stress at both the systemic and tissue level? This review aims to provide insight into these questions and highlight research questions that need to be answered in the future.

## 2. Oxidative Stress in Chronic Kidney Disease

**Figure 1:**
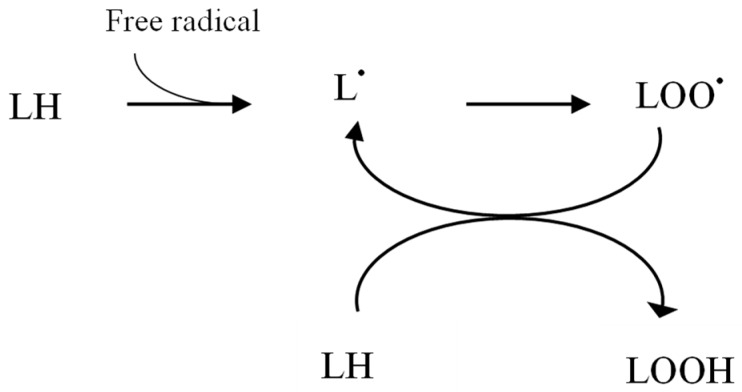
Reactive oxygen species (ROS)-induced lipid peroxidation. LH (lipid), LOOH (peroxide lipid), L^•^ (lipid radical), LOO^•^ (peroxidised lipid radical).

The mechanism behind the oxidative stress in patients with chronic kidney disease is complex and multifactorial and not fully elucidated [7]. Mainly, it results from an imbalance between oxidant–antioxidant processes causing a pro-oxidant state [8]. Factors including decreased levels of the antioxidants such as GSH, and ascorbic acid and enhanced ROS generation (arising from uraemic toxins) in CKD predispose patients to potential oxidative damage [9]. In addition, by-products of oxidative stress including peroxide lipid (see Figure 1) oxidise low density lipoproteins and lipid radicals activate immune cells including monocytes and macrophages leading to inflammation and further oxidative stress [10].

### 2.1. Increased Pro-Oxidant Activity

**Figure 2:**
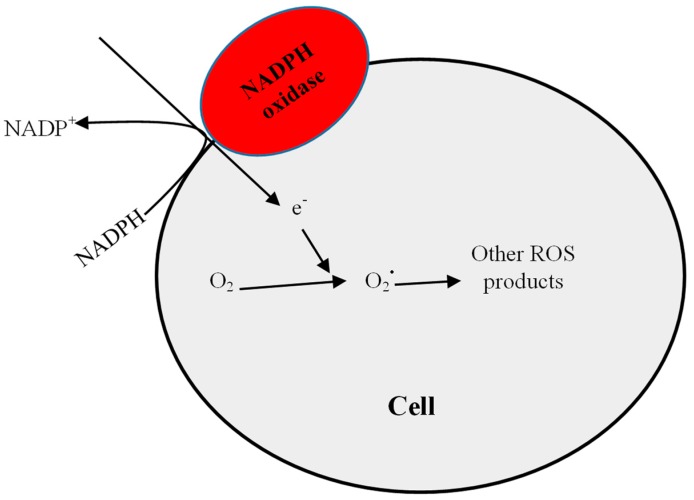
Schematic representation of NADPH oxidase-dependant ROS generation. NADPH under the influence of the plasma membrane-bound NADPH oxidase donates an electron, which is accepted by molecular oxygen to form the superoxide (O_2_^•^) radical.

Oxidative stress in uraemia may in part be due to increased pro-oxidant activities leading to excessive ROS production. This argument is supported by evidence of increased nicotinamide adenine dinucleotide phosphate (NADPH) oxidase activity or expression in models of renal dysfunction [11,12]. In diabetic nephropathy, elevated expression of renal NADPH oxidase and endothelial nitric oxide synthase (eNOS) resulted in oxidative stress with elevated lipid peroxidation which was inhibited following treatment with an angiotensin-converting enzyme inhibitor (ACEi) or an angiotensin receptor blocker (ARB) agent in rat models [13]. Increased expression of NADPH mediated an increased production of O_2_^•^ which was attenuated through treatment with apocynin (an inhibitor of NADPH oxidase) [14]. NADPH oxidases are a group of enzymes that reduce oxygen to O_2_^•^ radical (see Figure 2), which provides the starting point for the generation of other reactive oxidants including H_2_O_2_, lipid peroxides, and peroxynitrites (ONOO^•^) [15,16]. The NADPH oxidase family consists of five isoforms (Nox1 to Nox5) with p22phox as a bridge between the Nox family and the p47phox organizer factor [17,18]. Nox4 is expressed in vascular endothelial, smooth muscle cells, and renal proximal tubules [19,20], thus explaining why pathological insults involving the kidney affect its expression or activity.

Another possible source of ROS in CKD is xanthine oxidase whose activity can markedly increase in uraemia [21]. With molecular oxygen as the electron acceptor, xanthine oxidase catalyses the oxidation of hypoxanthine to uric acid, releasing ROS including O_2_^•^, OH^•^, and H_2_O_2_ as by-products. The metabolite uric acid is associated with CKD progression to renal failure and increased risk of cardiovascular events [22]. Thus, targeting xanthine oxidase as a way of lowering oxidative stress and uric acid levels (the increase of which causes severe joint pains such as gout) could be a putative therapeutic strategy in CKD. The observation that enhanced uptake of indoxyl sulphate by Nox4 via organic anion transporter 1 (OAT1) and 3 (OAT3) induces the production of O_2_^•^ radical [23] and supports the argument that uraemic toxins in CKD are associated with oxidative stress.

### 2.2. Uraemic Toxins

Impaired renal clearance in CKD leads to the accumulation of toxins of which *p*-cresol and indoxyl sulphate have been most widely studied [24]. Indeed, the uraemic toxin indoxyl sulphate confers an additional cardiovascular risk in CKD and upregulates the expression of intercellular adhesion molecule-1 (ICAM) and monocyte chemotactic protein-1 (MCP-1) [25]. It also induces the activation of NADPH oxidase and causes the production of ROS. *p*-Cresol sulphate, a conjugated form of *p*-Cresol, is reported to induce ROS in a time- and concentration-dependent manner [26]. Concentrations of indoxyl sulphate and *p*-Cresol sulphate have been shown to correlate inversely with GPx activity, indicating toxin-associated reduction in antioxidant capacity [27]. Indoxyl sulphate- and *p*-Cresol sulphate-mediated induction of oxidative stress are reported to occur through the activation of the nuclear factor-kappaB (NF-κB) pathway [28]. This mechanism was supported by in vivo experimental studies which demonstrated inhibition/reduction of oxidative stress following treatment with NF-κB inhibitors [29] and antioxidants [30]. Furthermore, a dose-dependent decrease in indoxyl sulphate through treatment with the drug AST-120 was found to significantly reduce oxidative stress in patients with CKD [31] and uraemic rats [32]. Oxidative stress-dependent loss of NO was alleviated in AST-120-treated CKD rats via the reduction of indoxyl sulphate [33]. This could explain the recently revealed benefits of AST-120 on the progression and prognosis of pre-dialysis CKD, including the reduction in the prevalence of dialysis requirement, mortality, and cardiovascular- and stroke-related events in treated patients after three and five years relative to untreated patients [34]. The accumulation of uraemic toxins in patients with CKD can also enhance inflammation, a potent inducer of oxidative stress [35,36].

### 2.3. Inflammation

**Figure 3:**
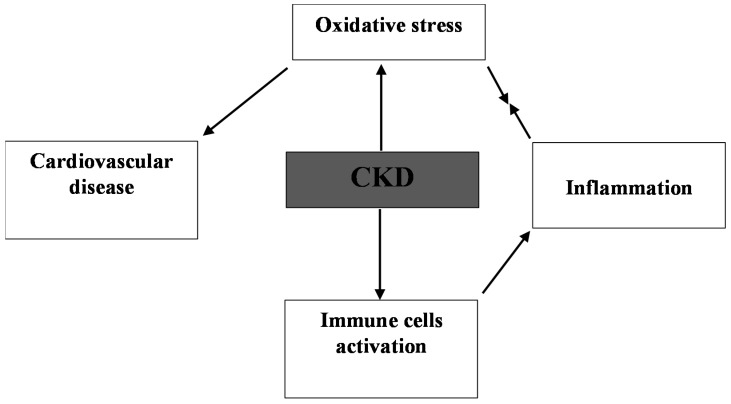
Risks of oxidative stress in CKD.

Patients with CKD are in a constant inflammatory state attributable to a number of factors, including the uraemic state, malnutrition, chronic volume overload, increased infection, metabolic acidosis, and autonomic dysfunction [37,38]. Inflammatory cells (e.g., phagocytes, monocytes/macrophages, or polymorphonuclear leukocytes) release reactive substances such as O_2_^•^ at the site of inflammation causing oxidative stress and ROS that, in turn, can initiate intracellular signalling cascade that activates pro-inflammatory gene expression [39]. For example, an increase in ROS is associated with the induction of inflammation, consequently increasing the levels of mediators such as interleukin-6 (IL-6) and tumour necrosis factor β (TGF-β) [40]. Thus, inflammation and oxidative stress in CKD are interlinked, with synergy between them magnifying the deleterious consequences associated with either of them alone (see Figure 3). Elevated concentrations of the pro-inflammatory markers C-reactive protein (CRP) and IL-6 significantly correlated with protein carbonyl (caused by oxidative damage to proteins) levels in early CKD [41]. The upregulation of inflammatory markers (e.g., tumour necrosis factor-α and platelet-derived growth factor) in CKD [42] are linked to NADPH oxidase activation leading to the generation of intracellular O_2_^•^ and H_2_O_2_ [43]. The inflammatory state in CKD can lead to activation/recruitment of polymorphonuclear neutrophils and monocytes, causing the activation of myeloperoxidase (MPO) and triggering ROS production [44].

### 2.4. Impaired Antioxidant System

Chronic deficiency in the antioxidant system, including the reduction of vitamin E, melatonin, SOD, catalase, and activity of glutathione system, has been reported in patients with kidney disease [45]. The reduction of SOD suggests the accumulation of O_2_^•^ radical causing increased lipid peroxidation [46]. The reduction of the endogenous thiol tripeptidic GSH antioxidant [47] together with the increased accumulation of GSSG due to the reduced GR activity [48] in patients with kidney disease further explain the prevalence of oxidative stress in this patient population. In addition, oxidative stress in CKD resulted from decreased catalase which compromised the conversion of H_2_O_2_ to H_2_O and O_2_ [44].

Eight isoforms of GPx enzymes (GPx1–GPx8) thus far have been recognised in mammals [49]. Only five (GPx1–GPx4 and GPx6) are found to contain selenium and are called seleno-GPx [50]. The kidney is increasingly found to be a major source of GPx3 [51], although GPx3 is also found in small concentrations in the liver, heart, and skeletal muscle. Thus, any kidney injury (acute and chronic) could affect GPx3 expression and total GPx activity. A reduction in GPx activity has been reported early during the development of CKD and continued to decrease with the severity of the disease [52]. This was in contrast to the observation that GPx activity negatively correlated with creatinine clearance in CKD stage 1–5, although the authors also reported a progressively decreased activity at two- and four-month follow-up in untreated patients with CKD at stage 3–5 [53]. Such discordance in the literature highlights the need for further investigation into the role of antioxidants such as GPx in CKD-related oxidative stress, a focus of our ongoing research. Decreased activity of SOD, catalase, and GPx with concomitant accumulation of nitrotyrosine were observed in the blood of chronic kidney failure patients [54]. Nitrotyrosine formation and other forms of ROS-mediated inactivation of nitric oxide (NO) may contribute to hypertension and its associated cardiac remodelling in uraemic patients as a result of inhibited vasodilation. Evidentially, hypertension and increased oxidative stress in the animal model of CKD were both attenuated by antioxidant therapy [55,56].

### 2.5. Anaemia and Oxidative Stress in CKD

**Figure 4:**
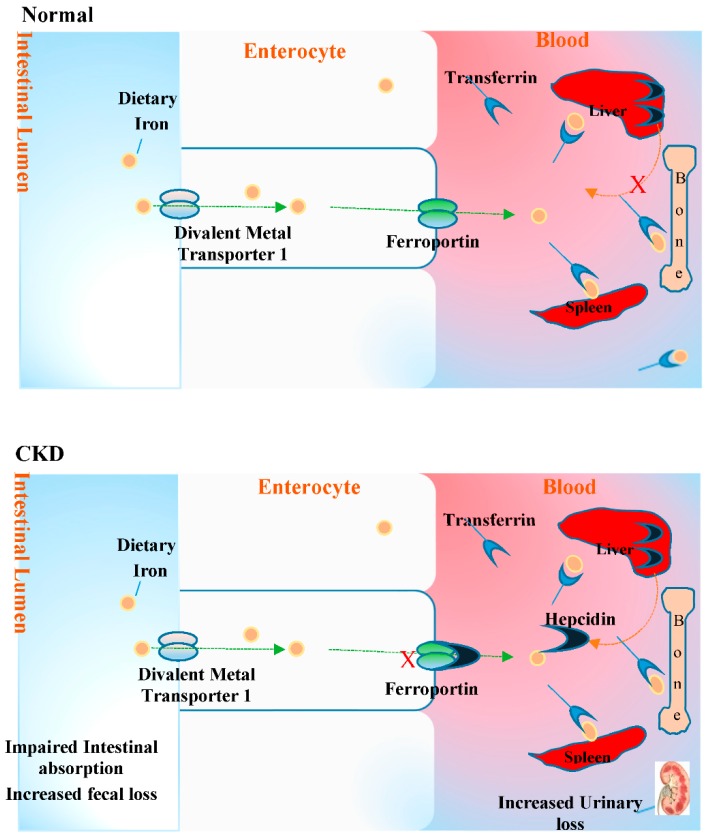
Mechanism of iron deficiency anaemia (IDA) in CKD. Normally, liver hepcidin synthesis is inhibited, allowing the uptake of dietary iron to replace the average 1–2 mg daily iron loss in adults via divalent metal transporter 1 (DMT1) at the apical brush border of enterocytes. Ferric reductase reduces the predominant Fe^3+^ to Fe^2+^ prior to DMT1 transport. Intracellular iron is stored as ferritin in the enterocytes while basolateral iron is transported by ferroportin into the circulation, where iron Fe^3+^ (generated by oxidation of Fe^2+^ by ferroxidases such as hephaestin) is loaded onto transferrin to be transported to storage (e.g., liver) or utilization site (bone marrow). In CKD, the presence of chronic inflammation with elevated cytokines such as IL-6 levels results in increased hepcidin secretion. Consequently, the binding of hepcidin to the iron transporter ferroportin induces its endocytosis and proteolytic destruction, thus preventing the transport of dietary iron into the circulation. Hepcidin secretion prevents the release of iron from storage sites [62]. Additionally, the apparent loss of iron via urine which is associated with kidney dysfunction may exacerbate IDA and reduce the efficacy of IV iron in the late stages of CKD.

Iron deficiency anaemia (IDA), a common complication in many chronic diseases including CKD, is caused by iron and erythropoietin deficiencies and a decreased responsiveness to the actions of erythropoietin [57,58]. Iron deficiency (ID) in CKD can be grouped into absolute and functional deficiency. Absolute iron deficiency in this patient group can be caused by low iron intake and complicated by impairment of dietary iron absorption in the gut, gastrointestinal bleeding, and increased blood loss [59]. Functional iron deficiency, on the other hand, is mediated by pro-inflammatory activation and hepcidin overproduction [60,61]. Hepcidin, which is produced by the liver, binds to and induces the degradation of ferroportin on hepatocytes, duodenal enterocytes, and reticuloendothelial macrophages, thereby inhibiting the absorption of dietary iron in the intestines and the release of iron from storage [62]. Hepcidin is also regulated transcriptionally by the inflammatory mediator IL-6 (known to be increased in CKD) via the STAT-3 signalling pathway [63,64]. Thus, enhanced inflammation in patients with CKD induces hepcidin production, causing iron sequestration and hypoferraemia and resulting in ID anaemia (see Figure 4) and oxidative stress [65].

Iron deficiency anaemia reduces the lifespan of red blood cells (RBCs) through increased premature RBC death [66] resulting from increased membrane stiffness and decreased deformability. The death of RBCs liberates iron which enhances the risk of oxidative stress. Oxidative stress itself can alter membrane properties of RBCs [67,68]. Indeed, exposure to H_2_O_2_ has been shown to increase lipid peroxidation and decrease cell deformability and membrane rigidity of erythrocytes [69], thereby increasing RBC removal in the spleen. This enhanced susceptibility of RBCs to oxidative damage [70] and the increased risk of ROS production in iron deficiency anaemia in CKD create a vicious cycle culminating in greater RBC death, anaemia, and severity of oxidative stress [71]. The accompanying decrease in haemoglobin content in ID anaemia lowers the partial pressure of oxygen similar to the hypoxic state. Under these conditions, hypoxia exacerbates oxidative stress through auto-oxidation of haemoglobin to met-haemoglobin (metHb) and with the generation of O_2_^•^ [72,73]. Endogenous antioxidant proteins such as catalase and peroxidase are iron-containing enzymes whose expression is affected in iron deficiency anaemia [74]. Iron deficiency adversely affects selenium concentration [75], which may explain the reduction in activity of the selenium-dependent enzyme GPx [76]. This evidence supports the argument that oxidative stress in iron deficiency anaemia is partly mediated via the reduction of antioxidant capacity [77] and suggests that timely iron replacement (via intravenous (IV) infusion rather than oral) and antioxidant therapies in clinical setting could lead to improvement. ID anaemia is associated with an adverse outcome in CKD; it certainly reduces the quality of life by increasing the risk of morbidity [78]. Hence, the management of anaemia and the treatment of its underlying cause, such as ID, must be a high priority.

Iron replacement can be done via oral, intravenous, or intramuscular routes, the former best given without food. Oral iron therapy is based on the premise that intestinal iron absorption is enhanced during iron deficiency and declines upon correction of the deficiency and replenishment of iron stores. Non-compliance among patients with CKD is complicated by the frequent side effects including nausea, constipation, diarrhoea, or abdominal pain associated with oral iron [79]. Moreover, oral iron therapy in advanced CKD is not recommended due to impaired intestinal iron absorption [80] as a result of increased hepcidin production. Examples of oral iron supplements include ferrous sulphate, ferrous fumarate, and ferrous gluconate. Unequivocally, IV iron therapy in CKD has been shown to be more efficacious than oral iron administration in stimulating haemoglobin synthesis and replenishing iron stores [81]. Intravenous iron regimes including iron dextran, iron sucrose, and ferric gluconate release bioactive iron associated with oxidative stress and inflammation [82]. There are mixed reports on the impact of IV iron on oxidative stress in patients with CKD. While some investigators associate IV iron with increased systemic oxidative stress [83], others see no effect [84]. It is increasingly becoming clear that the oxidative stress impact of IV iron is dependent on the iron formulation, the stage of CKD, the question of whether the patient is on dialysis, as well as the dose and time of investigation post-therapy [85].

Oxidative stress as evidenced by increased lipid and protein oxidation ensued following iron sucrose administration [86]. This side effect may be attributed to “free” (labile) iron levels which increase during iron therapy [87]. Free iron (biological active form) readily cycles between ferrous and ferric oxidation states. This property enables iron to aid the catalysis of biochemical reactions involved in the production of ROS, including hydroxyl radical, ferryl, and perferryl ion [88]. Hydroxyl radicals from the iron-catalysed Haber–Weiss reaction contribute to mitochondrial lipid peroxidation. Iron-induced lipid peroxidation is associated with intense mitochondrial damage [89] that may lead to a decline in oxidative capacity. Despite the well-documented acute and systemic oxidative toxicity of IV iron, not much is known of the long-term effect on the heart, skeletal muscle, and liver. Intravenous iron therapy however may enhance the risk of bacterial infection. Bacterial pathogens utilise iron to grow and increase virulence. Certain IV iron regimes such as ferric gluconate and iron sucrose reduce chemotaxis and phagocytosis of polymorphonuclear leukocytes, decreasing the immune response to bacterial infection [90]. However, the impact of IV iron therapy on cardiac mitochondrial function and mitochondrial oxidative stress in CKD is poorly defined, but given the central role of mitochondrial dysfunction in the development of heart failure, this may provide a major therapeutic strategy in limiting cardiovascular damage in CKD.

## 3. Consequence of Oxidative Stress in CKD

Oxidative stress in CKD can affect a number of organ systems with far-reaching clinical implications. It can both exacerbate kidney dysfunction and enhance progression and failure in other organs such as the heart, inducing cardiac hypertrophy which is an independent risk factor for heart failure (HF) and resulting in endothelial dysfunction. In considering the progression of CKD, part of the mechanism is via oxidant-induced damage to glomerular basement membrane [91,92]. Glomerular membrane integrity and thus glomerular ultrafiltration are compromised by oxidant-mediated impairment of glomerular heparin sulphate proteoglycans. Renal dysfunction is associated with the activation of the renin-angiotensin system (RAAS) [93], evidenced by the elevation of angiotensinogen (Ang) in CKD [94] and the increased release of renin in diabetic nephropathy [95,96]. RAAS activation is actively involved in the process of left ventricular (LV) remodelling partly through the induction of hypertension. Renin released in response to increased Na^+^ retention and local renal hypotension [97] converts angiotensinogen to angiotensin I (Ang I), which is further cleaved to give Ang II by the angiotensin converting enzyme (ACE). Ang II causes vasoconstriction and aldosterone release and consequently aggravates Na^+^ and water retention [98]. Thus, Ang II mediates haemodynamic alterations that culminate into cardiac and vascular remodelling. Therefore, the treatment of patients with LV dysfunction using ACE inhibitors such as captopril, enalapril, and Lisinopril has been associated with improved outcome and attenuation of ventricular enlargement [99].

Moreover, Ang II and aldosterone elicit non-haemodynamic effects that lead to the activation of mitogen-activated protein kinases (MAPKs), Ki-ras2A, and c-Src pathways involved in inflammation, ROS release (O_2_^•^ and H_2_O_2_), and endothelial dysfunction [100]. Ang II mediates the formation of O_2_^•^ via activation of NADPH oxidases [101]. This is supported by the finding of NADPH-mediated O_2_^•^ production following Ang II infusion [102]. Similarly, the treatment of rat aortic vascular smooth muscle cells (VSMCs) with Ang II increased intracellular generated O_2_^•^ by three-fold [103]. Increased O_2_^•^ resulted in increased protein kinase C (PKC) activity and NOS uncoupling causing impaired NO/cGMP signalling [104]. Thus, oxidative stress can cause loss of vasodilation, a hallmark of endothelial dysfunction. Cellular NO originates from NOS with tetrahydrobiopterin (BH4) as a cofactor. Excess ROS can oxidise BH4 which modifies NOS activity from NO generation to an O_2_^•^-producing oxidase [105], decreasing NO concentration and vasodilation. Endothelial dysfunction mediated by ONOO^•^-associated loss of vasodilation is implicated in hypertension, a unique contributor to hypertrophic remodelling [106].

Oxidative stress also leads to left ventricular hypertrophy (LVH) in CKD [107]. Thus, the co-existence of oxidative stress and LVH as independent complications of CKD further increases the risk of heart failure (HF) and cardiac events. ROS are pivotal in the initiation and propagation of signal transduction pathways, including tyrosine kinase Src, GTP-binding protein Ras, PKC, mitogen-activated protein kinase (MAPK), and Jun-nuclear kinase (JNK) implicated in hypertrophic growth [108]. Oxidative stress caused by elevated NADPH oxidase activity was associated with MAPK activation in a pressure overload model of LVH [109]. Similarly, NADPH oxidase-dependent generation of ROS in α1-adrenoreceptor-stimulated hypertrophy in adult rat ventricular myocytes (ARVMs) culture was associated with RAAS activation mediated through thiol oxidation [110]. The overexpression of thioredoxin-1 abolished the thiol oxidation and inhibited phosphorylation of MEK1/2 and ERK1/2, causing the inactivation of RAAS and the prevention of cellular hypertrophy.

Both chronic and acute renal dysfunction are unequivocally associated with cardiac abnormalities. In addition, mitochondrial targeted antioxidant therapy ameliorated cardiac hypertrophy and diastolic dysfunction via the attenuation of oxidative stress in experimental studies [111], further indicating the association between LVH and oxidative stress. Thus, antioxidant therapy in CKD could regress LVH and lessen progression to heart failure.

## 4. Cardio-Renal Oxidative Stress

Relative to the general population, patients with CKD have an additional risk of cardiovascular-related death [112]. Indeed, about 44–50% of deaths in the renal failure (RF) patient population were from cardiovascular causes [113], a mortality that is 15–30 times higher compared to the general population [114]. Likewise, congestive heart failure patients are associated with chronic kidney dysfunction such that every 1-mL/min decrease in creatinine clearance increases the mortality rate by 1% in this patient population [115]. These epidemiological data suggest synergistic effects between heart and kidney dysfunction resulting in poorer outcomes relative to either disease alone. This synergy gives rise to a clinical state known as cardio-renal syndrome. Cardio-renal syndrome (CRS) refers to the coexistence of both heart and kidney diseases, whereby dysfunction in one organ precipitates dysfunction in the other [116,117]. The unique interaction of the two clinical diseases (HF and RF) could in part be attributed to the distinct but interdependent function of the two organs and the existence of common causes, including hypertension and diabetes mellitus [118,119].

Invariably, oxidative stress in one organ induces injury in the other organ. The increased ROS associated with uraemia can induce cardiac injury because the exposure of rat ventricular myocytes to hydrogen peroxide resulted in cardiac remodelling with contractile dysfunction [120]. The role of oxidative stress in the induction of cardiac dysfunction in renal patients was highlighted in the Secondary Prevention with Antioxidants of Cardiovascular Disease in End-stage Renal Disease (SPACE) trial. In this study, antioxidant therapy using high dose vitamin E was associated with reduced cardiovascular disease endpoints in patients with kidney disease [121]. Moreover, increased NADPH oxidase mRNA and protein expression in HF rats has been observed along with increased accumulation of lipid peroxidation production and decreased endothelial NO synthase expression [122]. Conversely, renal oxidative stress is found in several models of CKD associated with cardiac dysfunction. In diabetic nephropathy, elevated expression of renal NADPH oxidase and eNOS resulted in oxidative stress with elevated lipid peroxidation, which was prevented following treatment with an angiotensin-converting enzyme inhibitor (ACEI) or an angiotensin receptor blocker (ARB) [13]. Similarly, diabetic nephropathy in Sprague Dawley rats was associated with increased glomerular expression of the NADPH oxidase subunit (p47phox), which is associated with enhanced oxidative stress [122,123,124].

Also, antioxidant therapy using the thiol-containing compound acetylcysteine reduced the risk of primary cardiovascular endpoint (including fatal and non-fatal myocardial infarction) by 40% in haemodialysis patients [125]. However, data from other clinical trials showed that antioxidant therapy was ineffective in patients with heart failure without renal disease [40]. These data support the argument that systemic oxidative stress in the uraemic milieu is central to the development of cardiac disease in renal patients as compared to non-renal HF patients. The disparity in the literature regarding the efficacy of antioxidant therapy on cardiovascular disease could partly be due to the differences in dosage, patient selection, and trial duration [40]. More importantly, this disparity highlights fundamental differences in the causes and effects of oxidative stress in cardio-renal patients and HF patients without renal dysfunction.

## 5. Future Questions

At what stage of CKD does remodelling of the oxidant–antioxidant system in the heart begin and how does systemic oxidative stress predict cellular derailment in both the heart and kidney? What associations exist between iron deficiency anaemia and mitochondrial dysfunction in CKD which is linked to oxidative stress? Ongoing work in our lab is focusing on providing insight into these important questions to allow us to develop therapies in the future.

## 6. Conclusions

Oxidative stress, anaemia, and LVH are complications in CKD which can individually contribute to the adverse cardiovascular outcomes in this patient population. There remains a need to discover viable and novel therapeutic regimes targeting the oxidative stress pathway early in CKD before the onset of significant irreversible renal failure.

## References

[1] Cachofeiro V., Goicochea M., Garca de Vinuesa S., Oubina P., Lahera V., Luno J. (2008). Oxidative stress and inflammation, a link between chronic kidney disease and cardiovascular disease. Kidney Int..

[2] Sinha N., Dabla P.K. (2015). Oxidative stress and antioxidants in hypertension—A current review. Curr. Hypertens. Rev..

[3] Blair A.I. (2008). DNA adducts with lipid peroxidation products. J. Biol. Chem..

[4] Kabel A.M. (2014). Free Radicals and Antioxidants: Role of Enzymes and Nutrition. World J. Nutr. Health.

[5] Panth N., Paudel K.R., Parajuli K. (2016). Reactive Oxygen Species: A Key Hallmark of Cardiovascular Disease. Adv. Med..

[6] Kao M.P.C., Ang D.S.C., Pall A., Struthers A.D. (2010). Oxidative stress in renal dysfunction: Mechanisms, clinical sequelae and therapeutic options. J. Hum. Hypertens..

[7] Dennis J.M., Witting P.K. (2017). Protective Role for Antioxidants in Acute Kidney Disease. Nutrients.

[8] Sung C.C., Hsu Y.C., Chen C.C., Lin Y.F., Wu C.C. (2013). Oxidative stress and nucleic acid oxidation in patients with chronic kidney disease. Oxid. Med. Cell. Longev..

[9] Vaziri N.D. (2004). Roles of oxidative stress and antioxidant therapy in chronic kidney disease and hypertension. Curr. Opin. Nephrol. Hypertens..

[10] Oberg B.P., McMenamin E., Lucas F.L., McMonagle E., Morrowm J., Ikizler T.A., Himmelfarb J. (2004). Increased prevalence of oxidant stress and inflammation in patients with moderate to severe chronic kidney disease. Kidney Int..

[11] Perianayagam M.C., Liangos O., Kolyadam A.Y., Wald R., MacKinnon R.W., Li L., Rao M., Balakrishnan V.S., Bonventre J.V., Pereira B.J. (2007). NADPH oxidase p22phox and catalase gene variants are associated with biomarkers of oxidative stress and adverse outcomes in acute renal failure. J. Am. Soc. Nephrol..

[12] Castilla P., Dávalos A., Teruel J.L., Cerrato F., Fernández-Lucas M., Merino J.L., Sánchez-Martín C.C., Ortuño J., Lasunción M.A. (2008). Comparative effects of dietary supplementation with red grape juice and vitamin E on production of superoxide by circulating neutrophil NADPH oxidase in hemodialysis patients. Am. J. Clin. Nutr..

[13] Onozato M.L., Tojo A., Goto A., Fujita T., Wilcox C.S. (2002). Oxidative stress and nitric oxide synthase in rat diabetic nephropathy: Effects of ACEI and ARB. Kidney Int..

[14] Thallas-Bonke V., Thorpe S.R., Coughlan M.T., Fukami K., Yap F.Y., Sourris K., Penfold S., Bach L.A., Cooper M.E., Forbesm J.M. (2007). Inhibition of NADPH oxidase prevents AGE-mediated damage in diabetic nephropathy through a protein kinase C-α-dependent pathway. Diabetes.

[15] Bryk D., Olejarz W., Zapolska-Downar D. (2017). The role of oxidative stress and NADPH oxidase in the pathogenesis of atherosclerosis. Postepy Hig. Med. Dosw..

[16] Schulz A.M., Terne C., Jankowski V., Cohen G., Schaefer M., Boehringer F., Tepel M., Kunkel D., Zidek W., Jankowski J. (2014). Modulation of NADPH oxidase activity by known uraemic retention solutes. Eur. J. Clin. Investig..

[17] Shimoishi K., Anraku M., Kitamura K., Tasaki Y., Taguchi K., Hashimoto M., Fukunaga E., Maruyama T., Otagiri M. (2007). An oral adsorbent, AST-120 protects against the progression of oxidative stress by reducing the accumulation of indoxyl sulfate in the systemic circulation in renal failure. Pharm. Res..

[18] Sirker A., Zhang M., Shah A.M. (2011). NADPH oxidases in cardiovascular disease: Insights from in vivo models and clinical studies. Basic Res. Cardiol..

[19] Sachse A., Wolf G. (2007). Angiotensin II-Induced Reactive Oxygen Species and the Kidney. J. Am. Soc. Nephrol..

[20] Flyvbjerg A., Denner L., Schrijvers B.F., Tilton R.G., Mogensen T.H., Paludan S.R., Rasch R. (2004). Long-term renal effects of a neutralizing RAGE antibody in obese type 2 diabetic mice. Diabetes.

[21] Choi J.Y., Yoon Y.J., Choi H.J., Park S.H., Kim C.D., Kim I.S., Kwon T.H., Do J.Y., Kim S.H., Ryu D.H. (2011). Dialysis modality-dependent changes in serum metabolites: Accumulation of inosine and hypoxanthine in patients undergoing hemodialysis. Nephrol. Dial. Transplant..

[22] Su X., Xu B., Yan B., Qiao X., Wang L. (2017). Effects of uric acid-lowering therapy in patients with chronic kidney disease: A meta-analysis. PLoS ONE.

[23] Oshima N., Onimaru H., Matsubara H., Uchida T., Watanabe A., Takechi H., Nishida Y., Kumagai H. (2015). Uric acid, indoxyl sulfate, and methylguanidine activate bulbospinal neurons in the rvlm via their specific transporters and by producing oxidative stress. Neuroscience.

[24] Evenepoel P., Meijers B.K., Bammens B.R., Verbeke K. (2009). Uremic toxins originating from colonic microbial metabolism. Kidney Int..

[25] Tumur Z., Shimizu H., Enomoto A., Miyazaki H., Niwa T. (2010). Indoxyl sulfate upregulates expression of ICAM-1 and MCP-1 by oxidative stress-induced NF-kappaB activation. Am. J. Nephrol..

[26] Watanabe H., Miyamoto Y., Honda D., Tanaka H., Wu Q., Endo M., Noguchi T., Kadowaki D., Ishima Y., Kotani S. (2013). P-Cresyl sulfate causes renal tubular cell damage by inducing oxidative stress through the activation of NADPH oxidase. Kidney Int..

[27] Rossi M., Campbell K.L., Johnson D.W., Stanton T., Vesey D.A., Coombes J.S., Weston K.S., Hawley C.M., McWhinney B.C., Ungerer J.P.J. (2014). Protein-bound Uremic Toxins, Inflammation and Oxidative Stress: A Cross-sectional Study in Stage 3–4 Chronic Kidney Disease. Arch. Med. Res..

[28] Meijers B.K.I., Evenepoel P. (2011). The gut-kidney axis: Indoxyl sulfate, *p*-cresyl sulfate and CKD progression. Nephrol. Dial. Transplant..

[29] Motojima M., Hosokawa A., Yamato H., Muraki T., Yoshioka T. (2003). Uremic toxins of organic anions up-regulate PAI-1 expression by induction of NF-kappaB and free radical in proximal tubular cells. Kidney Int..

[30] Yu M., Kim Y.J., Kang D.H. (2011). Indoxyl sulfate-induced endothelial dysfunction in patients with chronic kidney disease via an induction of oxidative stress. Clin. J. Am. Soc. Nephrol..

[31] Yamamoto S., Kazama J.J., Omori K., Matsuo K., Takahashi Y., Kawamura K., Matsuto T., Watanabe H., Maruyama T., Narita I. (2015). Continuous Reduction of Protein-Bound Uraemic Toxins with Improved Oxidative Stress by Using the Oral Charcoal Adsorbent AST-120 in Haemodialysis Patients. Sci. Rep..

[32] Taki K., Niwa T. (2007). Indoxyl sulfate-lowering capacity of oral sorbents affects prognosis of kidney function and oxidative stress in chronic kidney disease. J. Ren. Nutr..

[33] Tumur Z., Niwa T. (2008). An oral sorbent AST-120 increases renal NO synthesis in uremic rats. J. Ren. Nutr..

[34] Sato E., Tanaka A., Oyama J., Yamasaki A., Shimomura M., Hiwatashi A., Ueda Y., Amaha M., Nomura M., Matsumura D. (2016). Long-term effects of AST-120 on the progression and prognosis of pre-dialysis chronic kidney disease: A 5-year retrospective study. Heart Vessels.

[35] Chiu C.A., Lu L.F., Yu T.H., Hung W.C., Chung F.M., Tsai I.T., Yang C.Y., Hsu C.C., Lu Y.C., Wang C.P. (2010). Increased levels of total P-cresylsulphate and indoxyl sulphate are associated with coronary artery disease in patients with diabetic nephropathy. Rev. Diabet. Stud..

[36] Sun C.Y., Hsu H.H., Wu M.S. (2012). *p*-Cresol sulfate and indoxyl sulfate induce similar cellular inflammatory gene expressions in cultured proximal renal tubular cells. Nephrol. Dial. Transplant..

[37] DeFIlippi C.R., Herzog C.A. (2017). Interpreting Cardiac Biomarkers in the Setting of Chronic Kidney Disease. Clin. Chem..

[38] Ori Y., Bergman M., Bessler H., Zingerman B., Levy-Drummer R.S., Gafter U., Salman H. (2013). Cytokine secretion and markers of inflammation in relation to acidosis among chronic hemodialysis patients. Blood Purif..

[39] Taetzsch T., Levesque S., McGraw C., Bookins S., Luqa R., Bonini G.M., Mason P.R., Oh U., Block L.M. (2015). Redox regulation of NF-κB p50 and M1 polarization in microglia. Glia.

[40] Giam B., Kaye D.M., Rajapakse N.W. (2016). Role of Renal Oxidative Stress in the Pathogenesis of the Cardiorenal Syndrome. Heart Lung Circ..

[41] Oberg B.P., McMenamin E., Lucas F.L., McMonagle E., Morrow J., Ikizler T.A., Himmelfarb J. (2004). Increased prevalence of oxidant stress and inflammation in patients with moderate to severe chronic kidney disease. Kidney Int..

[42] Imig J.D., Ryan M.J. (2013). Immune and Inflammatory Role in Renal Disease. Compr. Physiol..

[43] Forbes J.M., Coughlan M.T., Cooper M.E. (2008). Oxidative Stress as a Major Culprit in Kidney Disease in Diabetes. Diabetes.

[44] Kalantar-Zadeh K., Brennan M.L., Hazen S.L. (2006). Serum myeloperoxidase and mortality in maintenance hemodialysis patients. Am. J. Kidney Dis..

[45] Zargari M., Sedighi O. (2015). Influence of hemodialysis on lipid peroxidation, enzymatic and non-enzymatic antioxidant capacity in chronic renal failure patients. Nephro-Urol. Mon..

[46] Tbahriti H.F., Kaddous A., Bouchenak M., Mekki K. (2013). Effect of Different Stages of Chronic Kidney Disease and Renal Replacement Therapies on Oxidant-Antioxidant Balance in Uremic Patients. Biochem. Res. Int..

[47] Gonzalez-Rico M., Puchades M.J., Garcia R., Saez G., Tormos M.C., Miguel A. (2006). Effect of oxidative stress in patients with chronic renal failure. Nefrologia.

[48] Niwa T., Tsukushi S. (2001). 3-Deoxyglucosone and AGEs in uraemic complications: Inactivation of glutathione peroxidase by 3-deoxyglucosone. Kidney Int..

[49] Song J., Yu Y., Xing R., Guo X., Liu D., Wei J., Song H. (2014). Unglycosylated recombinant human glutathione peroxidase 3 mutant from Escherichia coli is active as a monomer. Sci. Rep..

[50] Labunskyy M.V., Hatfield L.D., Gladyshev N.V. (2014). Selenoproteins: Molecular Pathways and Physiological Roles. Physiol. Rev..

[51] Avissar N., Ornt D.B., Yagil Y., Horowitz S., Watkins R.H., Kerl E.A., Takahashi K., Palmer I.S., Cohen H.J. (1994). Human kidney proximal tubules are the main source of plasma glutathione peroxidase. Am. J. Physiol..

[52] Kuchta A., Pacanis A., Kortas-Stempak B., Ćwiklińska A., Ziętkiewicz M., Renke M., Rutkowski B. (2011). Estimation of Oxidative Stress Markers in Chronic Kidney Disease. Kidney Blood Press Res..

[53] Papavasiliou E.C., Gouva C., Siamopoulos K.C., Tselepis A.D. (2005). Erythrocyte PAF-acetylhydrolase activity in various stages of chronic kidney disease: Effect of long-term therapy with erythropoietin. Kidney Int..

[54] Michea L., Villagrán A., Urzúa A., Kuntsmann S., Venegas P., Carrasco L., González M., Marusic E. (2008). Mineralocorticoid receptor antagonism attenuates cardiac hypertrophy and prevents oxidative stress in uremic rats. Hypertension.

[55] Vaziri N.D., Oveisi F., Ding Y. (1998). Role of increased oxygen free radical activity in the pathogenesis of uremic hypertension. Kidney Int..

[56] Kalk P., Godes M., Relle K., Rothkegel C., Hucke A., Stasch J.P., Hocher B. (2006). NO-independent activation of soluble guanylate cyclase prevents disease progression in rats with 5/6 nephrectomy. Br. J. Pharmacol..

[57] Goodnough L.T., Nemeth E., Ganz T. (2010). Detection, evaluation, and management of iron-restricted erythropoiesis. Blood.

[58] Sathyan S., George S., Vijayan P. (2017). Prevalence of anemia and cardiovascular diseases in chronic kidney disease patients: A single tertiary care centre study. Int. J. Adv. Med..

[59] Mehdi U., Toto R.D. (2009). Anemia, Diabetes, and Chronic Kidney Disease. Diabetes Care.

[60] Babitt J.L., Lin H.Y. (2012). Mechanisms of anemia in CKD. J. Am. Soc. Nephrol..

[61] Franchini M., Montagnana M., Lippi G. (2010). Hepcidin and iron metabolism: From laboratory to clinical implications. Clin. Chim. Acta.

[62] Ganz T., Nemeth E. (2012). Hepcidin and iron homeostasis. Biochim. Biophys. Acta.

[63] Pietrangelo A., Dierssen U., Valli L., Garuti C., Rump A., Corradini E., Ernst M., Klein C., Trautwein C. (2007). STAT3 is required for IL-6-gp130-dependent activation of hepcidin in vivo. Gastroenterology.

[64] Falzacappa M.V., Vujic S.M., Kessler R., Stolte J., Hentze M.W., Muckenthaler M.U. (2007). STAT3 mediates hepatic hepcidin expression and its inflammatory stimulation. Blood.

[65] David V., Martin A., Isakova T., Spaulding C., Qi L., Ramirez V., Zumbrennen-Bullough K.B., Sun C.C., Lin H.Y., Babitt J.L. (2016). Inflammation and functional iron deficiency regulate fibroblast growth factor 23 production. Kidney Int..

[66] Kempe D.S., Lang P.A., Duranton C., Akel A., Lang K.S., Huber S.M., Wieder T., Lang F. (2006). Enhanced programmed cell death of iron-deficient erythrocytes. Faseb. J..

[67] Malorni W., Straface E., Pagano G., Monti D., Zatterale A., Del Principe D., Deeva I.B., Franceschi C., Masella R., Korkina L.G. (2000). Cytoskeleton alterations of erythrocytes from patients with Fanconi’s anemia. FEBS Lett..

[68] Ghosh S., Bandyopadhyay S., Bhattacharya D.K., Mandal C. (2005). Altered erythrocyte membrane characteristics during anemia in childhood acute lymphoblastic leukemia. Ann. Hematol..

[69] Samanta S., Ghoshal A., Bhattacharya K., Saha B., Walden P., Mandal C. (2012). Sialoglycosylation of RBC in Visceral Leishmaniasis Leads to Enhanced Oxidative Stress, Calpain-Induced Fragmentation of Spectrin and Hemolysis. PLoS ONE.

[70] Snyder L.M., Fortier N.L., Trainor J., Jacobs J., Leb L., Lubin B., Chiu D., Shohet S., Mohandas N. (1985). Effect of hydrogen peroxide exposure on normal human erythrocyte deformability, morphology, surface characteristics, and spectrin-hemoglobin cross-linking. J. Clin. Investig..

[71] Vives-Corrons J.L., Miguel-Garcia A., Pujades M.A., Miguel-Sosa A., Cambiazzo S., Linares M., Dibarrart M.T., Calvo M.A. (1995). Increased susceptibility of microcytic red blood cells to in vitro oxidative stress. Eur. J. Haematol..

[72] Nagababu E., Gulyani S., Earley C.J., Cutler R.G., Mattson M.P., Rifkind J.M. (2008). Iron-deficiency anaemia enhances red blood cell oxidative stress. Free Radic. Res..

[73] Hebert P.C., Van der Linden P., Biro G., Hu L.Q. (2004). Physiologic aspects of anemia. Crit. Care Clin..

[74] Rifkind J.M., Nagababu E. (2013). Hemoglobin Redox Reactions and Red Blood Cell Aging. Antioxid. Redox Signal..

[75] Lazarte S.S., Mónaco M.E., Jimenez C.L., Achem M.E.L., Terán M.M., Issé B.A. (2015). Erythrocyte Catalase Activity in More Frequent Microcytic Hypochromic Anemia: Beta-Thalassemia Trait and Iron Deficiency Anemia. Adv. Hematol..

[76] Yetgin S., Hincal F., Basaran N., Ciliv G. (1992). Serum selenium status in children with iron deficiency anemia. Acta Haematol..

[77] Prats M., Font R., García C., Muñoz-Cortés M., Cabré C., Jariod M., Romeu M., Giralt M., Martinez-Vea A. (2014). Oxidative stress markers in predicting response to treatment with ferric carboxymaltose in nondialysis chronic kidney disease patients. Clin. Nephrol..

[78] Steinmetz H.T. (2012). The role of intravenous iron in the treatment of anemia in cancer patients. Ther. Adv. Hematol..

[79] Kumerova A., Lece A., Skesters A., Silova A., Petuhovs V. (1998). Anaemia and antioxidant defence of the red blood cells. Mater. Med. Pol..

[80] Kidney International KDIGO Clinical (2009). Practice Guidelines for the Diagnosis, Evaluation, Prevention and Treatment of Chronic Kidney Disease-Mineral and Bone Disorders. J. Int. Soc. Nephrol..

[81] Bhandari S. (2011). Beyond efficacy and safety-the need for convenient and cost-effective iron therapy in health care. NDT Plus.

[82] Hayat A. (2008). Safety Issues with Intravenous Iron Products in the Management of Anemia in Chronic Kidney Disease. Clin. Med. Res..

[83] Ganguli A., Kohli H.S., Khullar M., Lal Gupta K., Jha V., Sakhuja V. (2009). Lipid peroxidation products formation with various intravenous iron preparations in chronic kidney disease. Ren. Fail..

[84] Connor J., Butcher A. (2014). Evaluation of Serum Oxidative Stress Indices Following Intravenous Iron Delivery in Women with Iron Deficiency Anemia. Blood.

[85] Bailie G.R., Schuler C., Leggett R.E., Li H., Li H.D., Patadia H., Levin R. (2013). Oxidative effect of several intravenous iron complexes in the rat. Biometals.

[86] Swarnalatha G., Ram R., Neela P., Naidu M.U., Dakshina Murty K.V. (2010). Oxidative stress in hemodialysis patients receiving intravenous iron therapy and the role of N-acetylcysteine in preventing oxidative stress. Saudi J. Kidney Dis. Transpl..

[87] Schaller G., Scheiber-Mojdehkar B., Wolzt M., Puttinger H., Mittermayer F., Hörl W.H., Fodinger M., Sunder-Plassmann G., Vychytil A. (2005). Intravenous iron increases labile serum iron but does not impair forearm blood flow reactivity in dialysis patients. Kidney Int..

[88] Pietrangelo A. (2016). Mechanisms of iron hepatotoxicity. Hepatology.

[89] Almeida A.M., Bertoncini C.R.A., Boreky J., Souza-Pinto N.C., Vercesi A.E. (2006). Mitochondrial DNA damage associated with lipid peroxidation of the mitochondrial membrane induced by Fe2^+^-citrate. Ann. Braz. Acad. Sci..

[90] Bhandari S., Pereira D.I.A., Chappell H.F., Drakesmith H. (2018). Intravenous irons: From basic science to clinical practice. Pharmaceuticals.

[91] Brewster U.C., Perazella M.A. (2004). Intravenous iron and the risk of infection in end-stage renal disease patients. Semin. Dial..

[92] Diamond J.R. (1992). The role of reactive oxygen species in animal models of glomerular disease. Am. J. Kidney Dis..

[93] Takimoto E., Kass D.A. (2007). Role of oxidative stress in cardiac hypertrophy and remodeling. Hypertension.

[94] Siragy H.M., Carey R.M. (2010). Role of the Intrarenal Renin-Angiotensin-Aldosterone System in Chronic Kidney Disease. Am. J. Nephrol..

[95] Toma I., Kang J.J., Sipos A., Vargas S., Bansal E., Hanner F., Meer E., Peti-Peterdi J. (2008). Succinate receptor GPR91 provides a direct link between high glucose levels and renin release in murine and rabbit kidney. J. Clin. Investig..

[96] Kobori H., Alper A.B., Shenava R., Katsurada A., Saito T., Ohashi N., Urushihara M., Miyata K., Satou R., Hamm L.L. (2009). Urinary angiotensinogen as a novel biomarker of the intrarenal renin-angiotensin system status in hypertensive patients. Hypertension.

[97] Saito T., Urushihara M., Kotani Y., Kagami S., Kobori H. (2009). Increased urinary angiotensinogen is precedent to increased urinary albumin in patients with type 1 diabetes. Am. J. Med. Sci..

[98] Ruster C., Wolf G. (2009). Renin-angiotensin-aldosterone system and progression of renal disease. J. Am. Soc. Nephrol..

[99] Manrique C., Lastra G., Gardner M., Sowers J.R. (2009). The Renin Angiotensin Aldosterone System in Hypertension: Roles of Insulin Resistance and Oxidative Stress. Med. Clin. N. Am..

[100] Morrone D., Marzilli M. (2010). Role of RAAS inhibition in preventing left ventricular remodeling in patients post myocardial infarction. Heart Metab..

[101] Montezano A.C., Callera G.E., Yogi A., He Y., Tostes R.C., He G., Schiffrin E.L., Touyz R.M. (2008). Aldosterone and Angiotensin II Synergistically Stimulate Migration in Vascular Smooth Muscle Cells Through c-Src-Regulated Redox-Sensitive RhoA Pathways. Arterioscler. Thromb. Vasc. Biol..

[102] Cat A.N.D., Montezano A.C., Burger D., Touyz R.M. (2013). Angiotensin II, NADPH Oxidase, and Redox Signaling in the Vasculature. Antioxid. Redox Signal..

[103] Mollnau H., Wendt M., Szocs K., Lassegue B., Schulz E., Oelze M., Li H., Bodenschatz M., August M., Kleschyov A.L. (2002). Effects of angiotensin II infusion on the expression and function of NAD(P)H oxidase and components of nitric oxide/cGMP signaling. Circ. Res..

[104] Herbert K.E., Mistry Y., Hastings R., Poolman T., Niklason L., Williams B. (2008). Angiotensin II-mediated oxidative DNA damage accelerates cellular senescence in cultured human vascular smooth muscle cells via telomere-dependent and independent pathways. Circ. Res..

[105] Johnson R.J., Lovett D., Lehrer R.I., Couser W.G., Klebanoff S.J. (1994). Role of oxidants and protease in glomerular injury. Kidney Int..

[106] Taddei S., Virdis A., Ghiadoni L., Magagna A., Salvetti A. (1998). Vitamin C improves endothelium-dependent vasodilation by restoring nitric oxide activity in essential hypertension. Circulation.

[107] Paoletti E., Bellino D., Cassottana P., Rolla D., Cannella G. (2005). Left ventricular hypertrophy in nondiabetic predialysis CKD. Am. J. Kidney Dis..

[108] Cave A.C., Grieve D.J., Johar S., Zhang M., Shah A.M. (2005). NADPH oxidase-derived reactive oxygen species in cardiac pathophysiology. Philos. Trans. R. Soc..

[109] Li J.M., Gall N.P., Grieve D.J., Chen M., Shah A.M. (2002). Activation of NADPH oxidase during progression of cardiac hypertrophy to failure. Hypertension.

[110] Kuster G.M., Pimentel D.R., Adachi T., Ido Y., Brenner D.A., Cohen R.A., Liao R., Siwik D.A., Colucci W.S. (2005). Alpha-adrenergic receptor-stimulated hypertrophy in adult rat ventricular myocytes is mediated via thioredoxin-1-sensitive oxidative modification of thiols on Ras. Circulation.

[111] Dai D.F., Chen T., Szeto H., Nieves-Cintrón M., Kutyavin V., Santana L.F., Rabinovitch P.S. (2011). Mitochondrial targeted antioxidant peptide ameliorates hypertensive cardiomyopathy. J. Am. Coll. Cardiol..

[112] Alani H., Tamimi A., Tamimi N. (2014). Cardiovascular co-morbidity in chronic kidney disease: Current knowledge and future research needs. World J. Nephrol..

[113] Shah B.N., Greaves K. (2011). The Cardiorenal Syndrome: A Review. Int. J. Nephrol..

[114] Kon V., Yang H., Fazio S. (2015). Residual Cardiovascular Risk in Chronic Kidney Disease: Role of High-density Lipoprotein. Arch. Med. Res..

[115] McAlister F.A., Ezekowitz J., Tonelli M., Armstrong P.W. (2004). Renal insufficiency and heart failure: Prognostic and therapeutic implications from a prospective cohort study. Circulation.

[116] Ronco C., Haapio M., House A.A., Anavekar N., Bellomo R. (2008). Cardiorenal syndrome. J. Am. Coll. Cardiol..

[117] Ronco F., Ronco C. (2009). Cardiorenal syndrome, current understanding. Recenti Prog. Med..

[118] Rosner M.H., Ronco C., Okusa M.D. (2012). The role of inflammation in the cardio-renal syndrome: A focus on cytokines and inflammatory mediators. Semin. Nephrol..

[119] Small D.M., Gobe G.C. (2013). Oxidative Stress and Antioxidant Therapy in Chronic Kidney and Cardiovascular Disease. InTech.

[120] Josephson R.A., Silverman H.S., Lakatta E.G., Stern M.D., Zweier J.L. (1991). Study of the mechanisms of hydrogen peroxide and hydroxyl free radical-induced cellular injury and calcium overload in cardiac myocytes. J. Biol. Chem..

[121] Boaz M., Smetana S., Weinstein T., Matas Z., Gafter U., Iaina A., Knecht A., Weissgarten Y., Brunner D., Fainaru M. (2000). Secondary prevention with antioxidants of cardiovascular disease in endstage renal disease (SPACE): Randomised placebo-controlled trial. Lancet.

[122] Tojo A., Onozato M.L., Kobayashi N. (2002). Angiotensin II and oxidative stress in Dahl Salt-sensitive rat with heart failure. Hypertension.

[123] Kitada M., Koya D., Sugimoto T., Isono M., Araki S., Kashiwagi A., Haneda M. (2003). Translocation of Glomerular p47phox and p67phox by Protein Kinase C-β Activation Is Required for Oxidative Stress in Diabetic Nephropathy. Diabetes.

[124] De Blasio M.J., Ramalingam A., Cao A.H., Prakoso D., Ye J.M., Pickering R., Watson A.M.D., de Haan J.B., Kaye D.M., Ritchie R.H. (2017). The superoxide dismutase mimetic tempol blunts diabetes-induced upregulation of NADPH oxidase and endoplasmic reticulum stress in a rat model of diabetic nephropathy. Eur. J. Pharmacol..

[125] Tepel M., van der Giet M., Statz M., Jankowski J., Zidek W. (2003). The antioxidant acetylcysteine reduces cardiovascular events in patients with end-stage renal failure: A randomized, controlled trial. Circulation.

